# ASSOCIATION OF CARDIOMETABOLIC RISK FACTORS AND TRANSITION OF DISABILITY STATUS AMONG CHINESE ADULTS

**DOI:** 10.1093/geroni/igae098.3800

**Published:** 2024-12-31

**Authors:** Heming Pei, Gong Chen, Jiajia Li, Bo Liang

**Affiliations:** Columbia University, New York, New York, United States; Peking University, Beijing, Beijing, China; Peking University, Beijing, Beijing, China (People’s Republic); Peking University, Beijing, Beijing, China (People’s Republic)

## Abstract

**Background:**

Chronic diseases significantly increase the risk of functional disability. Understanding the relationship between cardiometabolic risk factor (CMRF) comorbidities and transitions in disability status is crucial for devising early intervention strategies at the population level.

**Method:**

This study utilized a prospective cohort design, drawing data from the China Health and Retirement Longitudinal Study (CHARLS) spanning from 2011 to 2018. A total of 6,397 Chinese middle-aged and older adults were followed for a total of 44,779 person-years. A Multi-state Markov model was employed to simulate longitudinal disability status transitions and to estimate the effect sizes of CMRFs, along with demographic, socioeconomic, and behavioral factors.

**Results:**

Over the 7-year follow-up, CHARLS participants experienced an increased likelihood of transitioning from normal physical function to IADL (Instrumental Activities of Daily Living) impairment, rising from 7.6% to 11.0%. Moreover, the transition intensity from intact ADL (Activities of Daily Living) to IADL impairment was 2.5 times higher than that from intact ADL to ADL impairment. Unlike other chronic conditions, CMRFs particularly influenced ADL by significantly reducing the probability of recovery from ADL impairment to a better physical function state. Specifically, the probability of shifting from IADL impairment to intact ADL decreased by 30% (HR=0.70, 95% CI [0.53-0.92]) among individuals with two CMRF components.

**Conclusion:**

The findings suggest that CMRFs act as risk factors by impeding the recovery process of ADL impairment. Early intervention targeting obesity, hypertension, hyperlipidemia, and diabetes could help reduce or delay the incidence of functional disability.

